# Numerical Study on Residual Stresses and Plastic Strains in Cold-Formed High-Strength Steel Circular Hollow Sections

**DOI:** 10.3390/ma16186337

**Published:** 2023-09-21

**Authors:** Ye Yao, Wai-Meng Quach

**Affiliations:** 1 School of Resources and Civil Engineering, Northeastern University, Shenyang 110819, China; yaoye@mail.neu.edu.cn; 2 Department of Civil and Environmental Engineering, University of Macau, Macau, China

**Keywords:** residual stresses, high-strength steel, circular hollow section, cold work, finite element modeling

## Abstract

This paper presents a numerical investigation on the residual stresses and co-existent equivalent plastic strains in cold-formed high-strength steel (CFHSS) circular hollow sections (CHS) by using an advanced finite element (FE)-based method. In this method, the entire manufacturing process of the CFHSS CHS was modeled numerically. The accuracy of the numerical predictions of equivalent plastic strains and residual stresses in the CFHSS CHS was verified by comparing the predictions with the existing test results of both the residual stress measurement and load-end shortening response of the stub column. By using the FE-based method, the effects of high-frequency electric resistance welding on the residual stresses and the stub column response were investigated. The through-thickness variations of both the equivalent plastic strains and residual stresses in CFHSS CHS, which are difficult to measure in the laboratory, were explored numerically. Finally, the effect of cold work (which is quantified by the equivalent plastic strains and residual stresses) on the stub column response of CFHSS CHS tubes was evaluated. It can be found that the equivalent plastic strains and longitudinal residual stresses are generally uniform around the cross-section of CFHSS CHS. The transverse and longitudinal residual stresses are generally uniform across each half-thickness, with the inner half-thickness under compression and the outer half-thickness under tension. The results also demonstrate that both the plastic strains and residual stresses may significantly affect the cross-section capacities of CFHSS CHS.

## 1. Introduction

High-strength steels (HSS) are available in the market and have been increasingly used in the construction of buildings, bridges, and tower structures, because of their advantages of high strength, low cost, and lightweight [1,2]. Cold-formed high-strength steel (CFHSS) tubular sections have been popular in the construction industry in recent years, due to their aesthetic appearance and outstanding structural performance [2,3]. However, the cold-forming process induces residual stresses and strength enhancement in cold-formed (CF) tubular sections, which may significantly influence their structural response. Therefore, an accurate determination of the residual stresses and co-existent equivalent plastic strains in CFHSS tubes becomes necessary. In addition, a better understanding of the effect of the residual stress and the co-existent equivalent plastic strains on the structural response of CF steel tubes is important as a key step in developing reliable design methods.

Residual stresses in CF steel tubes are generally evaluated by means of experimental studies, finite element (FE) simulations, theoretical predictions, or a combination of the aforementioned methods [4]. Early experimental investigations on the residual stresses of CF tubes were focused on carbon steels, as conducted by Kato and Aoki [5] for circular hollow sections (CHS) and by Key and Hancock [6] for square hollow sections (SHS). With the use of stainless steel becoming more popular in the construction industry, some studies have been conducted to determine residual stresses in stainless steel tubes, covering SHS and rectangular hollow sections (RHS) [7,8,9,10,11]. Existing experimental investigations on residual stresses in CFHSS tubes were scarce, and such measurements have been performed by Jiao and Zhou [12], Shi et al. [1], and Chen and Chan [13] for CHS; by Ma et al. [2] for SHS, RHS, and CHS; and by Liu et al. [14,15,16] for hexagonal, irregular hexagonal, and irregular octagonal hollow sections.

In the aforementioned experimental investigation of residual stresses in CFHSS tubes [1,2,12,13,14,15,16], only the surface residual stresses were measured as the tube thickness was small. From the previous experimental studies [17,18] on CF normal-grade steel tubes, it has been found that the distribution of residual stresses throughout the thickness is complex and difficult to measure. In addition, the relationships between each stage of the cold-forming process and residual stresses cannot be clearly established through the measurement results.

In addition to experimental studies, the theoretical solutions and FE simulations can also be used to predict the residual stresses in CF tubes. Kato and Aoki [5] developed the theoretical solutions for residual stresses in CF CHS with normal-grade steels by modeling its forming processes. Rossi et al. [19] simulated the forming processes for CFHSS and stainless steel channel sections by FE methods and investigated the strength enhancement in the corners of channel sections. Pastor et al. [20] investigated the residual stresses and initial imperfections in CF steel channel sections by modeling the forming process using FE simulations. However, studies on the theoretical solutions and FE modeling for determining residual stresses and plastic strains in CFHSS tubular sections are still scarce. Yao et al. [4,21] proposed an FE-based method to determine the strength enhancement and residual stresses in CF steel tubes and investigated residual stresses through the thickness in CF steel elliptical hollow sections (EHS), SHS, and RHS. So far, the through-thickness distributions of residual stresses and plastic strains in CFHSS CHS have not yet been studied in detail. Thus, the present study aims to bridge this research gap.

As mentioned earlier, it is difficult to measure residual stresses throughout the thickness accurately. Plastic strains in steel members cannot be measured directly. So, quantifying the effects of cold work (which are quantified by residual stresses and plastic strains) on both the material response and structural response of CFHSS CHS is challenging experimentally. Therefore, in the present study, an advanced FE-based modeling technique is presented to overcome these difficulties. In the present paper, the FE-based method proposed by Yao et al. [4,21] was first reviewed. Then, the FE-based method [4,21] was used to predict the residual stresses, plastic strains, and stub column behavior of CFHSS CHS. The accuracy of the FE predictions was verified by the existing test results of both the residual stress measurement and stub column response. By using this FE-based method [4,21], the effect of the welding process was investigated. The through-thickness variations of equivalent plastic strains and residual stresses in CFHSS CHS were investigated. Finally, the cold-work effect on the stub column response of CFHSS CHS was evaluated.

## 2. FE-Based Method

**Figure 1:**
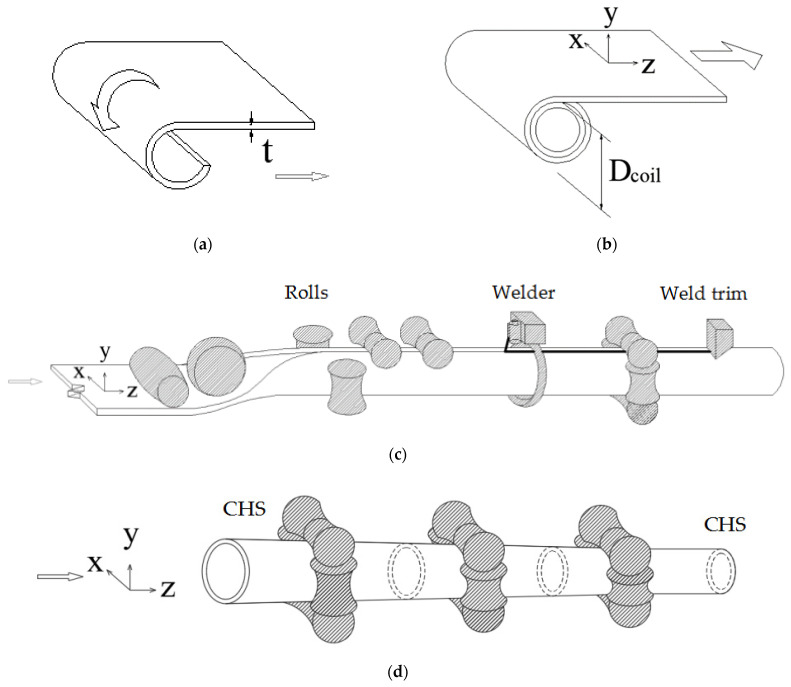
The manufacturing process of CF CHS: (**a**) coiling [4,22]; (**b**) uncoiling including flattening [4,22]; (**c**) transverse bending and welding operations [4,22]; and (**d**) shaping operation. (Reprinted from *Engineering Structures*, Vol 188, Yao Y., Quach W.M., Young B., Finite element-based method for residual stresses and plastic strains in cold-formed steel hollow sections, Figure 1, Pages No. 24–42, Copyright (2019), with permission from Elsevier. Reprinted from *Thin-Walled Structures*, Vol 154, Yao Y., Quach W.M., Young B., Simplified models for residual stresses and equivalent plastic strains in cold-formed steel elliptical hollow sections, Figure 1, Pages No. 106835, Copyright (2020), with permission from Elsevier).

A FE-based method has been presented in [4] to predict the equivalent plastic strains and residual stresses in CF tubes. Yao et al. [21] extended this FE-based method to predict the strength enhancement and the stub column behavior of CF steel tubes. The accuracy of the FE-based method [4,21] has been verified by the comparisons between the test results and FE predictions. In the FE-based method [4,21], the continuous-forming method, which is a commonly used manufacturing method for the production of CF tubes, was simulated. The entire continuous-forming process includes (1) the coiling–uncoiling operation (i.e., steel strips being coiled for storage and uncoiled into the production line of CF tubes), (2) the transverse bending from the uncoiled strip into a circular shape, (3) the welding along the longitudinal edges of the opened circular tube to form a closed tube, and (4) the shaping operation (i.e., shaping the welded circular tube into a desired shape, such as CHS, EHS, or RHS). The entire continuous-forming process is illustrated in Figure 1.

The modeling procedures of the FE-based method [4,21] involve five key stages, which are summarized below.

**Stage I: Analytical solutions for residual stresses induced by the coiling–uncoiling and transverse bending operations**

This stage aims to determine the equivalent plastic strains and residual stresses in the coiling–uncoiling and transverse bending operations, based on the analytical solutions derived by Quach et al. [23], Quach [24], and Quach and Cai [25]. The analytical predictions of equivalent plastic strains and residual stresses were then imposed in the FE model as initial states, for the subsequent FE simulations of welding and shaping.

**Stage II: FE simulation of welding**

The simulation of welding involves a heat transfer analysis and then a thermal stress analysis. The heat transfer analysis aims to determine the time-dependent temperature fields during heating (welding) and cooling processes. During the heat transfer analysis, heat conduction, cavity radiation, and heat convection were considered.

After the time-dependent temperature fields were obtained, these temperature fields were then imposed into the FE model of a subsequently coupled thermal stress analysis. The temperature-dependent mechanical properties needed for the thermal stress analysis were required in the FE analysis. By using the thermal stress analysis, the equivalent plastic strains and residual stresses due to the coiling–uncoiling, transverse bending, and welding operations can be obtained.

**Stage III: FE simulation of shaping**

The FE modeling of shaping was achieved by pulling the welded circular tube through desired rolls to become the final desired shape. In the FE simulation, the rolls were designed according to the current practical guidelines (see Section 3.5.1). The deformed FE mesh and its work-hardened states resulting from Stages I and II were imposed as the initial state in the FE model to simulate the shaping operation. The shaping operation was simulated by a general static stress analysis. Rolls were modeled as analytical rigid surfaces. Contact pairs were defined to consider the interaction between the steel tube and the rolls. For a cold-formed CHS, both the initial cross-section and final produced cross-section are still circular, but the perimeter and diameter of the final produced CHS tube become smaller than the initial size after shaping as a result of circumferential contraction (see Section 3.5.1).

From Stages I, II, and III, the equivalent plastic strains and residual stresses in CF tubes, caused by the coiling–uncoiling, transverse bending, welding, and shaping operations, can be determined.

**Stage IV: FE simulation of the sectioning operation in residual strain measurement**

The surface residual strains in CF tubes were often measured by the sectioning method. However, the sectioning operations may affect the measured results. Therefore, this stage aims to model the effect of the sectioning operation used in residual strain measurements on the obtained residual stresses. In this stage, the sectioning sequence was simulated according to the measurement procedures reported in laboratory measurements [2]. It means that the operation of cutting a short-length portion (which is approximately equal to a stub column length) from a long member was simulated. However, the procedure of cutting the short-length portion into longitudinal strips was not modeled. It was assumed that longitudinal strains in the stub column specimen were completely released after being sectioned into small strips. A further explanation of the simulation of sectioning is presented in Section 3.6.

**Stage V: FE modeling for the structural response**

The FE-based method for residual stresses (Stages I~IV) can be further extended to predict the structural response of CF steel tubes [21]. After the short-length portion was extracted (as described in Stage IV), the command *SYMMETRIC MODEL GENERATION in ABAQUS [26] was used to generate an entire stub column model. Then, the obtained stub column model with its work-hardened states and its imperfect mesh was subjected to axial loading in a new nonlinear buckling analysis to simulate the stub column behavior. Details about the entire modeling procedure of the FE-based method (Stages I–V) can be found in [4,21].

In this study, the equivalent plastic strains, residual stresses, and the structural response of CFHSS CHS were investigated based on the FE-based method [4,21] and are presented in the subsequent sections. The terminology and directions of stresses adopted in this paper should be noted first. The length direction of steel strips and tubes is defined as the longitudinal (*z*) direction and the width direction of the steel strip is defined as the transverse (*x*) direction, while the direction normal to the steel strip is defined as the through-thickness (*y*) direction. The location *y* = 0 is defined as the middle surface, while *y* = −*t*/2 is defined as the outer surface of the tube, where *t* is the sheet thickness.

## 3. FE Modeling for Residual Stresses and Stub Column Behavior

### 3.1. General

By using the FE-based method [4,21] (see Stages I–V in Section 2), the equivalent plastic strains, residual stresses, and stub column behavior of CFHSS CHS were investigated in this section. The existing experiments of CFHSS CHS (S139 × 6) conducted by Ma et al. [2,3] were selected to verify the FE model, in which the test results of both the residual stress and stub column behavior were provided. The measured outer diameter *D* and the thickness *t* of specimen S139 × 6 in [2,3] were 139.5 mm and 5.92 mm, respectively.

The modeling of material nonlinearity requires a virgin material’s true stress–true plastic strain relationship. The modeling of the mechanical behavior for virginal materials is presented in Section 3.2. The determination of the equivalent plastic strains and residual stresses in the coiling–uncoiling and transverse bending operations for CFHSS CHS is presented in Section 3.3. The FE simulation of welding operation for CFHSS CHS (including the necessary thermal and thermal–mechanical properties) is presented in Section 3.4. The FE simulation of shaping operation for CFHSS CHS (including the roll design for CHS) is presented in Section 3.5. In addition, the FE simulations of the sectioning operation and the stub column behavior of CFHSS CHS are presented in Section 3.6 and Section 3.7, respectively.

According to the suggestions of Yao et al. [4,21], the S4R shell element was selected in the thermal stress and static stress analyses, and the DS4 shell element was selected in the heat transfer analysis. Seventeen integration points were adopted [4,21]. The length of the FE model should be long enough to minimize the end effect and was suggested to be twice the longitudinal spacing of forming passes [4,21]. In this study, a modeled length of 1000 mm was selected.

**Figure 2:**
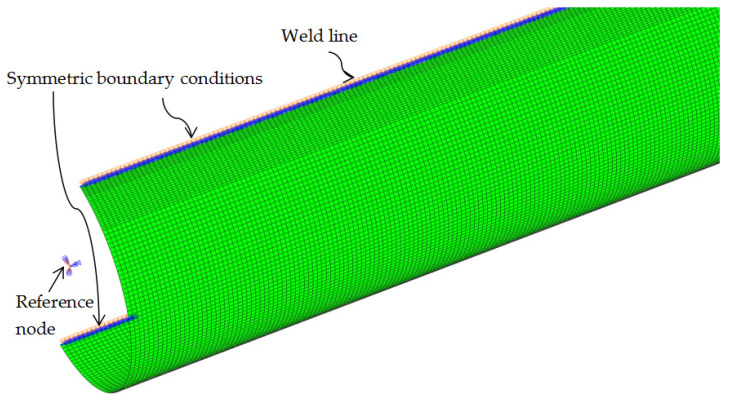
Half-section model that considers welding.

In the present study, the effect of welding on residual stresses and stub column behavior in CFHSS CHS was examined by comparing the predictions obtained from two different FE models: (1) the model that considers the welding (e.g., Stages I–V in Section 2 of this paper) and (2) the model that does not consider the welding (Stages I, III, IV, and V in Section 2). One-half of the section was modeled as the cross-section is symmetrical about the weld (see Figure 2).

A mesh convergence study has been conducted to obtain the final meshes. The adopted mesh consists of smaller elements near the weld (approximately 2.5 mm × 0.8 mm (length by width)) and larger elements for other portions (2.5 mm × 2.4 mm), as shown in Figure 2.

### 3.2. Virgin Material Properties at the Ambient Temperature

In the present study, a bilinear true stress $\sigma_{t}$–true strain $\varepsilon_{t}$ relationship, as given in Equation (1), was used to construct the virgin material $\sigma_{t}$–$\varepsilon_{t}$ curve for the $\sigma_{t}\leq \sigma_{tu}$ (where $\sigma_{tu}$ is true ultimate stress), according to the suggestions of Yao et al. [4,21] for HSS. For $\sigma_{t}\geq \sigma_{tu}$, the Quach and Qiu’s model [27] was used.

(1) $$\sigma_{t}=\left\{\begin{array}{ll} E_{0}\varepsilon_{t} & {,}_{}0\leq \varepsilon_{t}\leq \varepsilon_{ty}\\ \left(\frac{\sigma_{tu}-\sigma_{ty}}{\varepsilon_{tu}-\varepsilon_{ty}}\right)\left(\varepsilon_{t}-\varepsilon_{ty}\right)+\sigma_{ty} & {,}_{}\varepsilon_{ty}<\varepsilon_{t}\leq \varepsilon_{tu} \end{array}\right.$$

in which $E_{0}$ is the initial elastic modulus, and $\sigma_{ty}$ is the true yield stress of the virgin material, which is approximately equal to the corresponding engineering value $\sigma_{y,v}$; $\varepsilon_{ty}=\sigma_{ty}/E_{0}$; $\varepsilon_{tu}$ is the true ultimate stress. The values of $E_{0}$, $\sigma_{ty}$, $\sigma_{tu}$, and $\varepsilon_{tu}$ are the input in this stress–strain model.

As measured virgin material properties were not available for the considered CFHSS CHS in [2], the $E_{0}$ was assumed to be 210 GPa based on the EC3-defined value for carbon steels [28]. It has been found that the considered CFHSS CHS was fabricated from hot-rolled sheets or plates of grades Raex 400 (manufactured by RUUKKI, Helsinki, Finland), as indicated in its mill certificates [29,30]. As virginal steel materials, the typical yield stress of Raex 400 was 1000 MPa. Therefore, the value of $\sigma_{y,v}$ was assumed to be 1000 MPa, based on its typical yield stress.

The values of $\sigma_{tu}$ and $\varepsilon_{tu}$ were determined based on the ultimate strain $\varepsilon_{u,c}$ and ultimate stress $\sigma_{u,c}$ of their corresponding cold-worked materials, as shown in the following equations, based on the developments in [4,21]:

(2) $$\sigma_{tu}≅\sigma_{tu,c}=\left(1+\varepsilon_{u,c}\right)\sigma_{u,c}$$

(3) $$\varepsilon_{tu}≅\ln\left(1+\varepsilon_{u,c}\right)+\varepsilon_{av,c}$$

**Figure 3:**
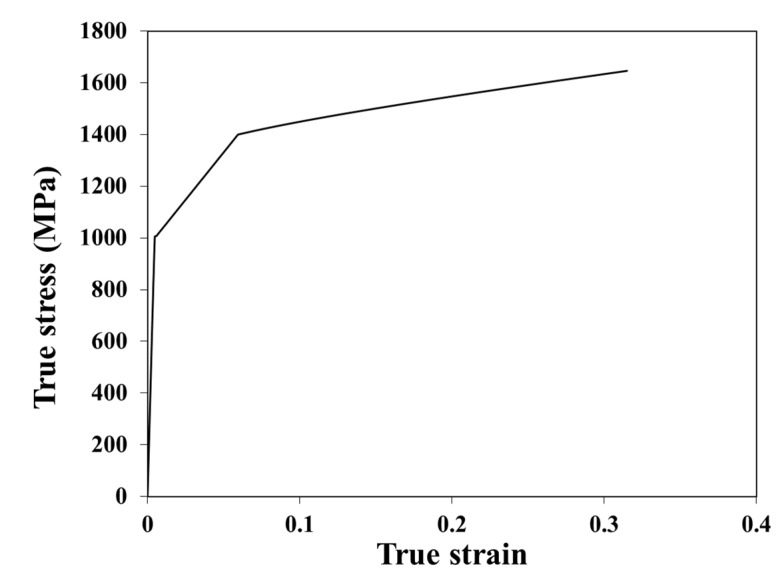
Virgin material true stress–strain curve of CFHSS CHS.

In Equation (3), $\varepsilon_{av,c}$ represents the through-thickness averaged plastic strain of cold-worked materials and its value can be given by Equation (4) for CF steel CHS [21]:

(4) $$\varepsilon_{av,c}=\frac{t}{2R_{coiling}}+\frac{0.5t}{2R_{CHS}}+\varepsilon_{\mu }\;\mathrm{with}\;\varepsilon_{\mu }=\mu -1$$

where $R_{coiling}$ is the coiling radius which was set to be 100*t* [4,21]; $R_{CHS}$ is the initial circular tube radius at the mid-plane surface; $\varepsilon_{\mu }$ is the plastic strains induced by the circumferential contraction; μ is the contraction ratio and its value was assumed to be 1.02 (see Section 3.5.1 for a further explanation). The values of $\varepsilon_{u,c}$ and $\sigma_{u,c}$ were measured by Ma et al. [2], which were equal to 1372 MPa and 0.02, respectively. The calculated $\varepsilon_{av,c}$, $\sigma_{tu}$, and $\varepsilon_{tu}$ were equal to 0.039, 1400 MPa, and 0.059, respectively. The complete virgin material $\sigma_{t}$–$\varepsilon_{t}$ curve of the considered CFHSS CHS is given in Figure 3.

### 3.3. Analytical Solutions for Residual Stresses Due to the Coiling–Uncoiling and Transverse Bending Operations

**Figure 4:**
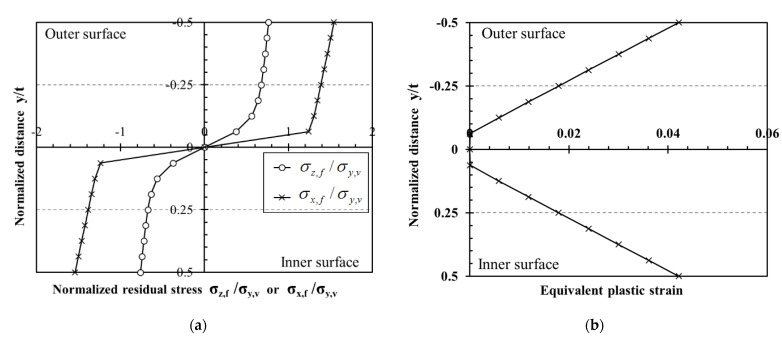
Stress states after coiling, uncoiling, and transverse bending operations for the CFHSS CHS: (**a**) residual stresses and (**b**) equivalent plastic strains.

Based on the virgin material properties provided in Section 3.2, the equivalent plastic strains and residual stresses in Stages I and II were determined based on analytical solutions derived by Quach and Cai [25]. Figure 4 shows the transverse residual stresses $\sigma_{x,f}$, longitudinal residual stresses $\sigma_{z,f}$, as well as the equivalent plastic strains ${\bar{\varepsilon }}_{p,f}$ across the thickness of a circular tube resulting from Stages I and II before it was shaped into the CFHSS CHS. In Figure 4, when y≤0, the $\sigma_{z,f}$ and $\sigma_{x,f}\geq 0$ as in tension.

### 3.4. FE Simulation of Welding

#### 3.4.1. Assumptions

The process of welding is complicated and is influenced by many parameters. The residual stress distributions induced by welding have been investigated by many researchers [31]. It should be noted that the purpose of the present study was to evaluate the effect of the residual stresses and the co-existed plastic strains on the buckling behavior of CFHSS CHS. As Hübner et al. [32] found that the buckling response was not sensitive to the welding-induced highly localized stresses, a simplified temperature history approach [32,33] was often adopted to determine the welding residual stresses for such a purpose. This approach only considers the cooling process after welding, which needs to input a welding-induced temperature field. Therefore, in this study, this temperature history approach [32,33] was used but considers both the cooling and heating processes.

For the CFHSS CHS considered in this study, high-frequency electric resistance welding (ERW) was adopted in the manufacturing process (see Figure 1). In the ERW, the strip edges are pressed together, by heating the steels to the melting temperature [34]. The strip edges are heated for a time duration $t_{h}$. Komine et al. [34] proposed an expression to calculate the heating time $t_{h}$ in the ERW, which is given by [4]:

(5) $$t_{h}=\frac{L_{w}}{v_{w}}$$

where $v_{w}$ is the welding speed ranging from 10 to 150 m/min [35]; and $L_{w}$ is the heating length. The simplified assumptions made by Komine et al. [34] have been used here for the FE model, which can be referred to [4] and briefly summarized as follows: (1) the $L_{w}$ in Equation (5) was equal to the outer diameter of the welded circular tube [35]; (2) the $v_{w}$ was equal to 10 m/min, which is the lower bound of the welding speed [36]; (3) the annealing effect was considered in the FE model.

#### 3.4.2. Heat Input

In the heat transfer analysis, the ambient temperature (20 °C), maximum (melting) temperature, and heating time are required in the FE model to determine the time-dependent temperature field. In this study, the melting temperature was set to be 1725 °C [37] during the heating process (i.e., welding) according to the common practice for high-frequency electric welding. The obtained heating time for the considered CFHSS CHS in this study was equal to 0.83 s by using Equation (5). In this study, the annealing temperature was assumed to be 800 °C [38].

#### 3.4.3. Thermal and Mechanical Properties at Elevated Temperatures

The mechanical and thermal properties at elevated temperatures are required in the FE model. The temperature-dependent thermal conductivity $\lambda_{a}$ for HSS was determined by the model proposed by Choi et al. [39]. The specific heat $c_{a}$ and thermal elongation $\Delta L/L_{0}$ (and thus the thermal expansion coefficient α) were determined by using the Eurocode 3 [40], as Choi et al. [39] have shown that the Eurocode-3 models for these two thermal properties are also applicable to high-strength steels.

For the considered CFHSS CHS, the stress–strain relations at elevated temperatures of its virginal material were determined by using the two-stage model for HSS proposed by Chen and Young [41] together with new retention factors proposed by Li and Young [42]. Chen and Young’s model [41] is defined by using the elastic modulus, 0.2% proof stress, ultimate stress, and ultimate strain at elevated temperatures and has been developed based on hot-rolled HSS sheet BISPLATE 80 with measured 0.2% proof stress of 789 MPa at the ambient temperature. Recently, Li and Young [42] proposed new retention factors for the elastic modulus, 0.2% proof stress, and ultimate stress of high-strength steels with a wide range of nominal yield stresses (from 690 MPa to 960 MPa); they found that the retention factor for the ultimate strain proposed by Chen and Young [41] can generally provide conservative predictions. Therefore, these retention factors [41,42] were adopted in this study. Other parameters needed in the FE models of welding can be referred to [4].

### 3.5. FE simulation of Shaping

#### 3.5.1. Design of Rolls

**Figure 5:**
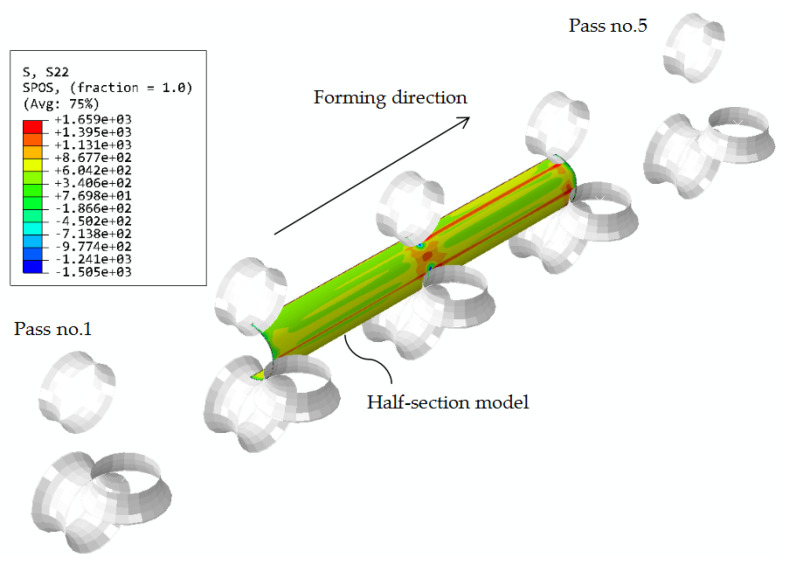
Stress contours (MPa) of a CFHSS CHS during the shaping operation (with the consideration of welding).

In general, each roll set consists of one bottom, one top, and two side rolls [43,44,45]. Three to five passes have been usually adopted [43,44,45]. In this study, five passes were adopted according to the suggestions of Yao et al. [4] (see Figure 5). The spacing between two adjacent passes was 500 mm, as suggested by Yao et al. [4]. The outer perimeter of a steel tube is gradually reduced due to the circumferential contraction by the rolls.

A circumferential contraction ratio μ was used to quantify the total amount of the contraction, as defined by Equation (6) [4,46,47]:

(6) $$\mu =\frac{P_{1}}{P_{n}}$$

where $P_{n}$ is the outer perimeter of the final shape, and $P_{1}$ is the outer perimeter of the initial circular shape. A contraction ratio $\bar{\mu }$ was used to quantify the contraction between two adjacent passes, as defined by Equation (7) [4,46,47]:

(7) $$\bar{\mu }=\frac{P_{i}}{P_{i+1}}$$

where $P_{1}$ is the outer perimeter of an intermediate shape. The value of $\bar{\mu }$ is given by Equation (8) [4,46,47]:

(8) $$\bar{\mu }=\mu^{1/\left(n-1\right)}$$

where *n* is the pass number. Therefore, for the given values of μ, *n*, and $P_{n}$, the outer perimeter $P_{i}$ can be calculated by using Equations (6)–(8).

The recommended values of μ for CF tubes range from 1.02 to 1.04 [46,47]. By trial and error, it was found that a μ value of 1.02 for the CFHSS CHS specimen led to the closest match between measured results and FE predictions. Therefore, the μ value of 1.02 was applied to the CFHSS CHS considered in this study.

**Figure 6:**
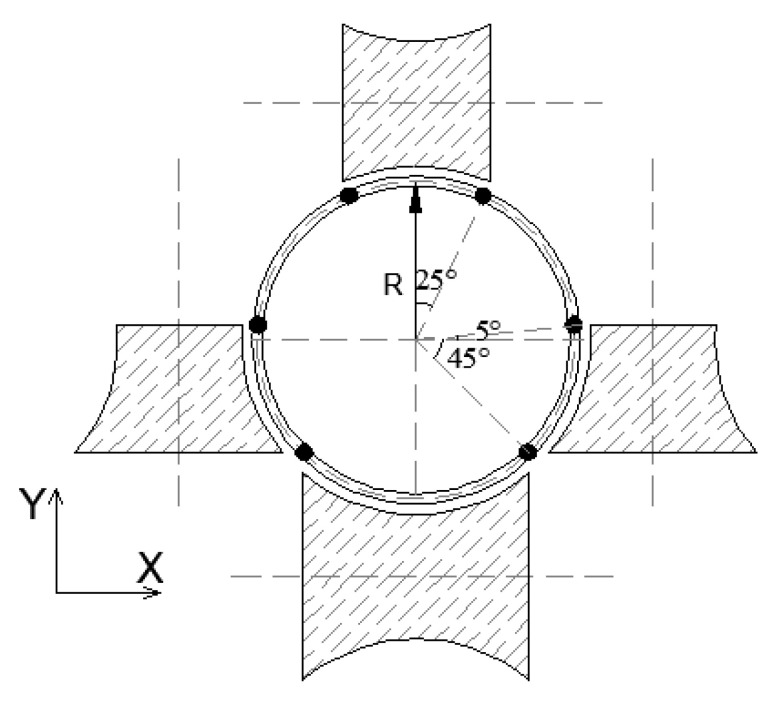
Roller design of the CHS.

As observed by Ma et al. [2], peak residual strains and greater strength enhancement have been found at several discrete locations, such as the locations at distances approximately equal to 30 mm and 100 mm from the weld. This may be attributed to the local cold bending at these locations. This observation suggests that these locations are due to either a local concentration caused by roll edges or a local bending. Therefore, the dimensions of the rolls have been designed by these locations. The roll design for the CFHSS CHS is illustrated in Figure 6.

#### 3.5.2. Static Stress Analysis

The deformed FE mesh with its work-hardened states resulting from Stages I, II, and III was imposed as the initial state for the FE shaping simulation. For the FE model that does not consider the welding effect, equivalent plastic strains and residual stresses due to Stages I and II were directly imposed as the initial state in the FE shaping simulation.

The FE model of the shaping operation for the CFHSS CHS is shown in Figure 5. A reference point was specified at the existing end of the tube to guide the tube through these passes. Details of the modeling procedure can be found in [4].

### 3.6. FE Simulation for Sectioning Operations

The effect of the sectioning operation on residual-strain measurements was considered in the FE simulation. According to the experimental procedure [2], the tube was first cut into a short specimen (e.g., 300 mm), and the strain gauges were attached to the outer surface and inner surface of the tube. The specimens were subsequently cut into longitudinal strips (e.g., approximately 10 mm). Strain measurements were taken before and after cutting the specimen.

**Figure 7:**
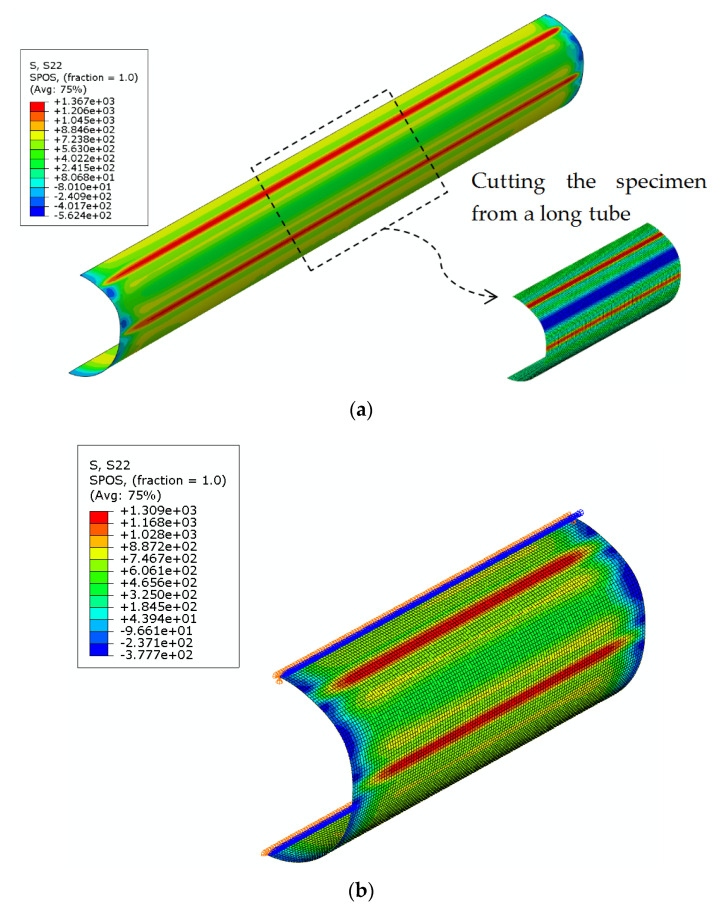
Stress contours (MPa) of CFHSS CHS at different steps of the FE simulation for the cutting procedure (without the consideration of welding): (**a**) initial state of a long tube and (**b**) after removal of the end.

In the FE analysis, only the operation of cutting the short specimen was simulated, the longitudinal strains in the short specimen were assumed to be completely released after sectioning into small strips. The modeling of the cutting operation is given in Figure 7. In this FE analysis, restraints on the nodes at both ends of the extracted short specimen were removed to simulate the cutting procedure. At the end of this simulation, the elastic strains in the longitudinal direction at the mid-length section of the short-length FE model were obtained and compared with the experimental measurements.

### 3.7. FE Modeling for the Stub Column Response in CFHSS CHS

**Figure 8:**
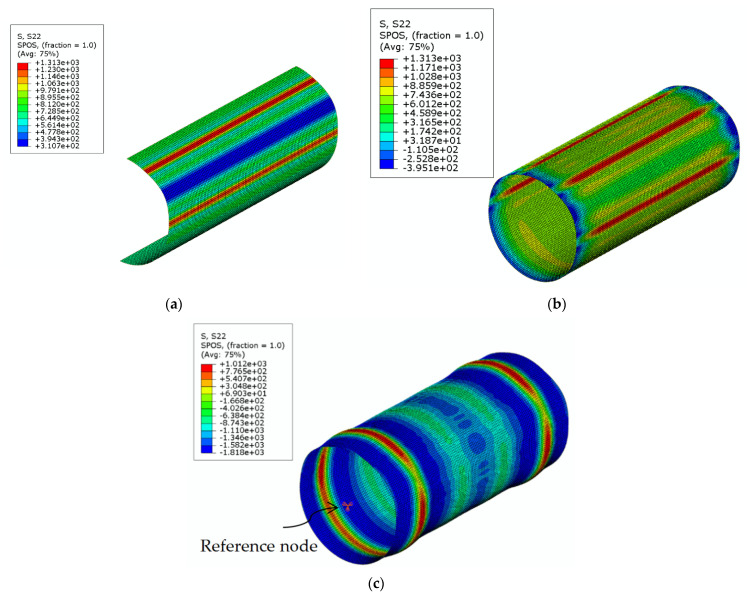
Stress contours (MPa) of CFHSS CHS at different steps of the FE simulation for stub column behavior (without the consideration of welding): (**a**) stress state of the stub column part in the long tube (half-section model); (**b**) stress state of the stub column (removal of end restrains); and (**c**) failure mode of the stub column after axial compression.

The FE-based method for residual stresses [4] was extended to predict the stub column behavior [21], as reviewed in Section 2 (Stage V). As shown in Figure 8, after the short length was extracted, a stub column model with the complete cross-section was generated by using the ABAQUS (Version 6.14) command *SYMMETRIC MODEL GENERATION [26] (see Figure 8a,b). Then, the simulation of cutting the stub column was conducted, by removing all the restraints on all nodes (see Figure 8c) to release the residual stresses and associated deformations after cutting.

By using the modified Riks method, the obtained stub column model with imperfect FE mesh was subjected to axial loading, to obtain the load-end shortening curve of the stub column. All degrees of freedom (DOF) of the nodes at each end of the column were constrained to the rigid body motion of a reference point located at the centroid of the column end (see Figure 8c). All the DOFs of the reference points were restrained except for the axial translation of one reference point to apply an axial compressive load.

## 4. Comparison of FE Predictions with Test Results

### 4.1. Residual Strains

**Figure 9:**
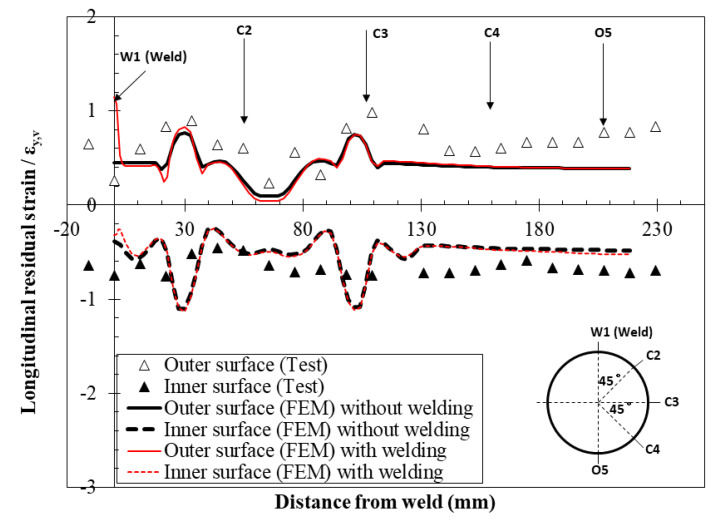
Comparison of surface residual strain distributions in the CFHSS CHS tested by Ma et al. [2] between measurements and FE predictions.

Figure 9 shows the comparison of FE predictions with measured longitudinal residual strains at the surface in CFHSS CHS. As shown in Figure 9, the residual strain (“+” is tension) is normalized by the $\varepsilon_{y,v}$. In Figure 9, the predictions from the two FE models with and without considering the effect of welding, respectively, were also compared to investigate the effect of high-frequency ERW on residual stresses. As the operation of high-frequency ERW is an intermediate process in the entire fabrication process, the test results in [2] cannot directly reflect the effect of ERW on residual stresses. Nevertheless, from the comparison in Figure 9, it can be found that the difference between the residual strains predicted by these two FE models was very small (only a little difference near the welding seam). Therefore, it can be concluded that the effect of high-frequency ERW on residual stresses in CFHSS CHS is negligible. FE-predicted residual strains are in close agreement with the measured results, which demonstrates that the FE-based method in [4] can be used to predict the residual stresses of CFHSS CHS.

### 4.2. Stub Column Behavior

**Figure 10:**
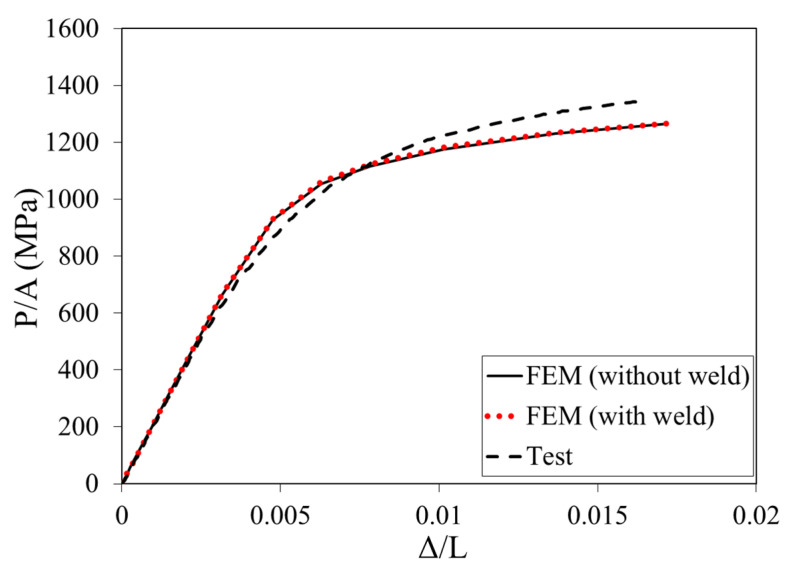
Comparison of the stub column behavior of the CFHSS CHS tested by Ma et al. [3] between test results and FE predictions.

Figure 10 shows the comparison of FE predicted load-end shortening curve with test results. In Figure 10, the load-end shortening curve from the FE model without considering the welding effect is also included. As shown in Figure 10, the P, *A*, Δ, and *L* are the load, cross-sectional area, end shortening, and length of a stub column, respectively. As shown in Figure 10, the predictions from the FE models with and without considering the effect of welding, respectively, were also compared to investigate the effect of high-frequency ERW on the stub column response. From the comparisons in Figure 10, it can be found that the difference between the load-end shortening curves predicted by these two models was very small (i.e., the two FE curves almost coincided). Therefore, it can be concluded that the effect of high-frequency ERW on the stub column response of CFHSS CHS is negligible. This can be explained that the high-frequency ERW-induced residual stresses and plastic strains were concentrated on a very small region, as shown in Figure 9. This localized effect has a negligible influence on the stub column behavior; this finding is also consistent with the conclusions of Hübner et al. [32], where Hübner et al. [32] found that the buckling response was not sensitive to the welding-induced highly localized stresses. The FE-predicted load-end shortening curves matched well with the test results, which can demonstrate that the FE-based method [4,21] can be adopted to predict the stub column response of CFHSS CHS. It is worth noting that the FE models slightly underestimated the ultimate loads. This is because the FE models take into account only the four major operations in the entire continuous-forming method. These four major operations are simplified and sorted out to be the most important parts of a complex forming process. In the production line of tube forming, there are also some other minor operations which may lead to additional cold work and thus enhance the ultimate load of stub columns. Therefore, the FE models may underestimate the ultimate strengths of stub columns (leading to slightly conservative strength predictions). As the stub column response of CF tubes is influenced by the cold-worked induced plastic strains and residual stresses, a good agreement of the load-end shortening curves between test results and FE predictions can also reflect that the FE-based method can accurately predict the cold-worked effect of CFHSS CHS.

## 5. Distributions of Equivalent Plastic Strains and Residual Stresses

**Figure 11:**
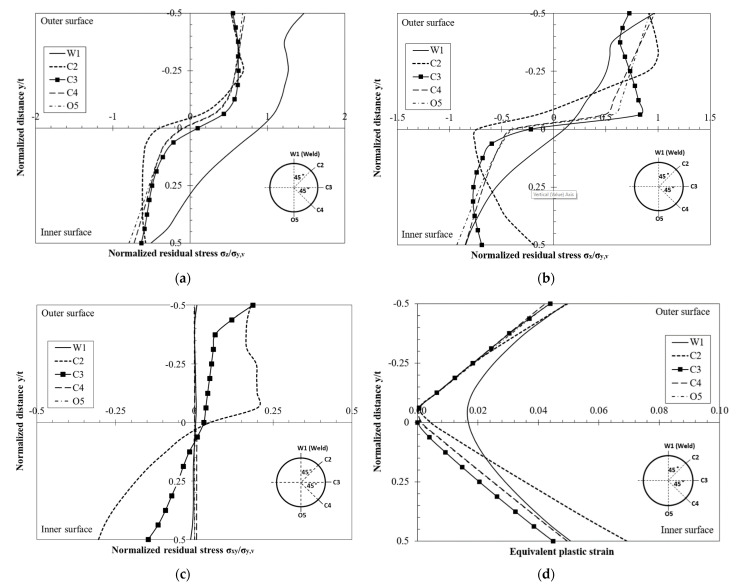
Through-thickness variations of residual stresses and equivalent plastic strains in the CFHSS CHS: (**a**) longitudinal residual stresses; (**b**) transverse residual stresses; (**c**) in-plane shear stresses; and (**d**) equivalent plastic strains.

From the comparisons of the FE-predicted residual stresses and stub column behavior with test results, as presented in Section 4, it has been concluded that the FE-based method [4,21] can be used to investigate the residual stress distribution of CFHSS CHS. It should be noted that the surface residual strain measurement may not provide detailed information about the plastic strains and residual stresses. For example, the through-thickness variations of residual stresses cannot be obtained by the measurement, and the plastic strains cannot be directly measured. Therefore, residual stresses and equivalent plastic strains in the tube thickness were investigated by using the FE model [4,21]. In order to eliminate the end effect, the FE results are taken at the mid-length section of the FE long column model. Figure 11 shows the through-thickness variations of both equivalent plastic strains and residual stresses at critical locations around the cross sections, and residual stresses are normalized by $\sigma_{y,v}$.

As shown in Figure 11, longitudinal, transverse residual stresses and equivalent plastic strains in CFHSS CHS were generally uniform around the circular cross-section, except the portion near the weld seam (point W1). Equivalent plastic strains at inner and outer surfaces (i.e., peak values) were approximately equal to 0.05 (see Figure 11d). It can be seen that the residual stresses in the outer half-thickness were mainly in tension, while those in the inner half-thickness were mainly in compression (Figure 11a,b). Both transverse and longitudinal residual stresses were fairly uniform across each half-thickness. The magnitudes of these uniform tensile and compressive residual stress blocks across the half-thicknesses were almost equal. This was due to the fact that the bending residual stresses in CFHSS CHS were predominant and the membrane residual stresses were relatively small. This conclusion was also found in the experimental results reported by Ma et al. [2]. The magnitude of longitudinal residual stresses was approximately equal to 0.6 $\sigma_{y,v}$, while that of transverse residual stresses was approximately equal to 0.8 $\sigma_{y,v}$. The magnitude of in-plane shear stresses was negligible (see Figure 11c), except for those at a few discrete locations (e.g., point C2). As these discrete regions (i.e., locations at and near point C2) were subjected to the local cold-bending caused by the edges of rolls, the in-plane shear stresses in these regions were more obvious than the other regions.

## 6. Effect of Cold Work on the Structural Response

In CF steel hollow sections, residual stresses are always induced together with the co-existent plastic strains (i.e., residual stresses in CF sections do not appear alone without plastic straining). Thus, the effect of residual stresses on the structural behavior of CFHSS CHS should be evaluated together with their plastic strains. In order to examine the importance of cold work, the effects of residual stresses and plastic strains on the stub column behavior of CFHSS were assessed. Four different CFHSS CHS specimens were selected according to the specimens in [3]. The sizes of these four specimens are as follows: (1) *D* = 139.0 mm with *t* = 6 mm; (2) *D* = 133.0 mm with *t* = 4 mm; (3) *D* = 108.0 mm with *t* = 4 mm; and (4) *D* = 89.0 mm with *t* = 4 mm. The stub column length was taken as 3 × *D* [3]. The steel grade of all these four specimens was Raex 400 [29,30], while the $E_{0}$ was assumed to be 210 GPa. The mechanical properties of virgin materials were also given in Section 3.2.

Two different FE stub column models were developed here to investigate the effect of cold work on the stub column behavior: (1) the stub column model with residual stresses and plastic strains and (2) the stub column model without residual stresses and plastic strains. In the stub column model with cold work, the plastic strains and residual stresses predicted by the FE-based method, which was adopted in this paper, were incorporated into the FE stub column model. In the stub column model without cold work, the residual stresses and plastic strains were not incorporated into the FE mesh. The local imperfections in these two FE stub column models were modeled by using the lowest eigenmode as the imperfection shape. The local geometric imperfection magnitude was set to be 0.2*t*, based on the model for CF CHS in [48].

**Figure 12:**
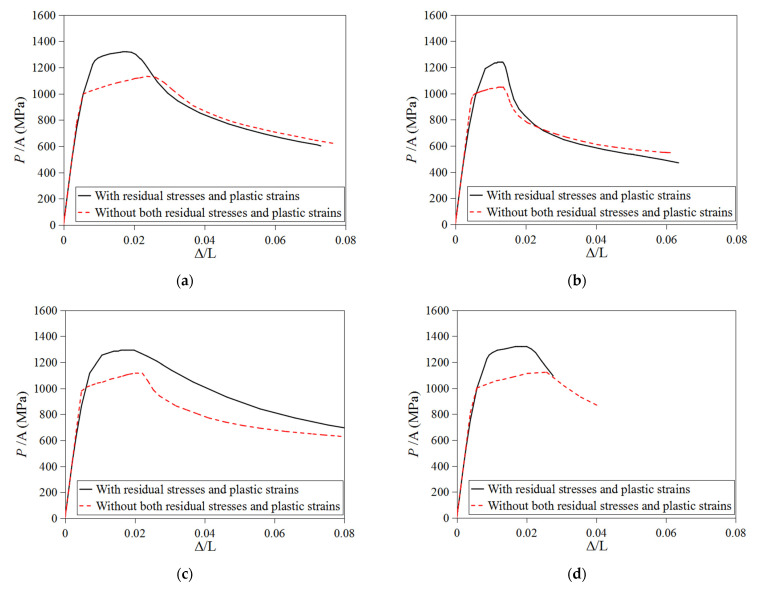
Effects of plastic strains and residual stresses on the stub column behavior of CFHSS CHS: (**a**) specimen 139 × 6; (**b**) specimen 133 × 4; (**c**) specimen 108 × 4; and (**d**) specimen 89 × 4.

Figure 12 shows the comparison of the load-end shortening curves predicted by the two FE stub column models on the aforementioned four CFHSS CHS specimens. As shown in Figure 12, both residual stresses and plastic strains caused a negative effect on the 0.2% proof stresses of stub columns, but their effect on the ultimate load of stub columns was not small. As a result of the plastic strains induced by cold forming, the ultimate loads of stub columns were significantly enhanced by the cold work. Therefore, it can be concluded that the effect of both residual stresses and plastic strains induced by the cold-forming process may significantly affect the cross-section capacities of CFHSS CHS. Thus, the evaluations of residual stresses and plastic strains are necessary for the investigation of the column behavior of CFHSS CHS.

## 7. Conclusions

In this paper, the residual stresses and equivalent plastic strains in CFHSS CHS were investigated by using the finite element (FE)-based method proposed by Yao et al. [4,21]. The FE predictions fit well with the test results of both residual stresses and stub column response, which can demonstrate that the FE-based method [4,21] can be applied to investigate the residual stresses and equivalent plastic strains of CFHSS CHS. By using the FE-based method [4,21], the predicted residual stresses and equivalent plastic strains in the tube thickness, which were difficult to measure in the laboratory, were investigated. The effects of welding on residual stresses and stub column response were investigated. Finally, the effect of cold work on the cross-section behavior of CFHSS CHS was also investigated. Major conclusions are given below:

(a) As the difference between the residual strains predicted by the two FE models (one considering the ERW and the other one without considering the ERW) was very small; it can be concluded that the effect of high-frequency ERW on residual stresses in CFHSS CHS is negligible in the continuous-forming process.
(b) In CFHSS CHS, the equivalent plastic strains and longitudinal residual stresses are generally uniform around the circular cross-section. The transverse and longitudinal residual stresses are generally uniform across each half-thickness, with the inner half-thickness under compression and the outer half-thickness under tension.
(c) As the bending residual stresses are predominant and the membrane residual stresses are relatively small in CFHSS CHS, the magnitudes of these uniform tensile and compressive residual stress blocks across the half-thicknesses are almost equal.
(d) In CFHSS CHS, the through-thickness variations of equivalent plastic strains are generally bilinear; the minimum value is near the middle surface of the wall thickness; and the peak values are at the inner and outer surfaces.
(e) The effect of both the plastic strains and residual stresses induced by the cold-forming process may significantly affect the cross-section capacities of CFHSS CHS. The ultimate load of stub columns can be highly enhanced as a result of the cold forming.

## Data Availability

Data available on request.
